# What’s Shape Got to Do With It? Examining the Relationship Between Facial Shape and Orofacial Clefting

**DOI:** 10.3389/fgene.2022.891502

**Published:** 2022-05-03

**Authors:** Seth M. Weinberg

**Affiliations:** ^1^ Department of Oral and Craniofacial Sciences, Center for Craniofacial and Dental Genetics, University of Pittsburgh, Pittsburgh, PA, United States; ^2^ Department of Human Genetics, University of Pittsburgh, Pittsburgh, PA, United States; ^3^ Department of Anthropology, University of Pittsburgh, Pittsburgh, PA, United States

**Keywords:** face shape, cleft lip, cleft palate, craniofacial anomalies, multifactoral threshold

## Abstract

Nonsyndromic orofacial clefts belong to a class of congenital malformations characterized by a complex and multifactorial etiology. During early facial development, multiple factors can disrupt fusion leading to a cleft; this includes the shape of the embryonic face. The face shape hypothesis (FSH) of orofacial clefting emerged in the 1960s, influenced by morphological differences observed within affected families, comparative studies of mouse models, and advances in modeling genetic liability for complex traits in populations. For the past five decades, studies have documented changes in the shape or spatial arrangement of facial prominences in embryonic mice and altered post-natal facial shape in individuals at elevated risk for orofacial clefting due to their family history. Moreover, recent studies showing how genes that impact facial shape in humans and mice are providing clues about the genetic basis of orofacial clefting. In this review, I discuss the origins of the FSH, provide an overview of the supporting evidence, and discuss ways in which the FSH can inform our understanding of orofacial clefting.

## Introduction

It is well established that nonsyndromic orofacial clefts have a complex multifactorial etiology (Garland et al., 2020; Leslie, 2021). Genes, endogenous and exogenous environmental factors, interactions among genes and/or environmental agents, and epigenetic modifications are all believed to play a role. These factors can lead to clefts though different mechanisms (Jiang et al., 2006). For example, the primary deficiency may involve the fusion process. In such cases, the facial prominences may grow in a normal manner and make contact, but something interferes with the molecular machinery that drives tissue fusion (reviewed in Ji et al., 2020). However, another possibility is that the nascent facial prominences are not able to contact one another or meet too late for proper fusion to take place. This implies that the tissues are in principle capable of fusing, but never get the opportunity. One way this could happen is an intrinsic deficiency in the growth of one or more of the facial prominences (e.g., due to hypoplasia). Another is that the potential for cellular proliferation is normal, but the trajectory of facial prominence growth may be abnormal. Yet another possibility is that an extrinsic force (e.g., brain growth) displaces the facial prominences in a way that interferes with their positions relative to one another. The key principle uniting all the above scenarios is that the precise spatial arrangement required for the facial prominences to meet and fuse is disrupted leading to a cleft. Put another way, the above scenarios point to alterations in biological shape, either of individual facial prominences or of the entire developing facial complex. The idea that shape can act as a precipitating factor in the emergence of an orofacial cleft is the fundamental premise of the face shape hypothesis (FSH). In this review I discuss the origins of, lines of supporting evidence for, and future perspectives on the FSH of orofacial clefting.

## Origins of the Face Shape Hypothesis

Three lines of converging evidence led to the development of the FSH: the first is observational and relates to the presence of distinctive orofacial traits in individuals whose baseline genetic risk for clefting is higher than average; the second is experimental and relates to work done using embryos of mouse strains with varying natural susceptibility to clefting; and the third is theoretical and relates to the establishment of models to conceptualize disease liability in populations.

The familial pattern of orofacial clefts was recognized at least as early as the 18^th^ century (Trew, 1757). With no formal knowledge of genetics, 19^th^ century surgeons suspected that parents with cleft affected children were passing on this hereditary tendency and claimed they could see the manifestation of this tendency in their faces (Fergusson, 1870; Rose, 1891). Studies documenting minor defects or asymmetries in the oronasal region of otherwise-healthy relatives (parents and/or siblings) from families with a history of orofacial clefting first surfaced in the 1930’s (Mengel, 1939) and again in the 1960’s (Fukuhara and Saito, 1962; Fukuhara and Saito, 1963; Rusconi and Brusati, 1966; Mills et al., 1968; Niswander, 1968). Likewise, Dixon (1966) noted a strong tendency toward a class III skeletal relationship (relative maxillary retrusion) in the parents of children with orofacial clefts. Many of these studies lacked proper controls and methods of phenotypic assessment were crude, but they established an important principle: those at genetically higher risk for orofacial clefting, by means of their familial connections, tend to exhibit a pattern of facial traits that seem to be in some way related to cleft predisposition.

As early as the late 1950’s, Fraser and colleagues noted that the rate of cortisone-induced cleft palate differed according to mouse strain and that several factors, including dimensions of the developing murine palate and head, can influence risk of spontaneous cleft palate (Fraser et al., 1957). A decade later, Trasler directly explored the possibility of facial shape as a predisposing factor (Trasler, 1968). In this work, Trasler qualitatively compared the faces of embryos from mouse strains with high and low rates of spontaneous clefting of the primary palate prior to fusion. More susceptible mice were characterized by differences in the relative position and shape of the medial nasal processes, such that the probability of contact with the adjacent lateral nasal and maxillary process was reduced. Since Trasler published her seminal findings, a number of subsequent studies have corroborated and expanded upon these observations by comparing the facial morphology and development of mouse strains with varying degrees of susceptibility to spontaneous clefting (Smiley et al., 1971; Juriloff and Trasler, 1976; Sulik et al., 1979; Trasler and Machado, 1979; Diewert, 1982; Millicovsky et al., 1982; Trasler and Ohannessian, 1983; Ciriani and Diewert, 1986; Jacobson and Trasler, 1992; Wang and Diewert, 1992). These studies point to alterations in the size/shape of individual facial prominences and/or their spatial arrangement relative to one another as being a factor in the lead up to failed fusion. More recently, studies applying modern morphometric methods and advanced imaging have found that susceptible mouse strains exhibit differences in facial shape (both in embryos and as adults), have less morphologically integrated faces, and show excess facial shape variation (Hallgrímsson et al., 2004; Young et al., 2007; Parsons et al., 2008).

**FIGURE 1:**
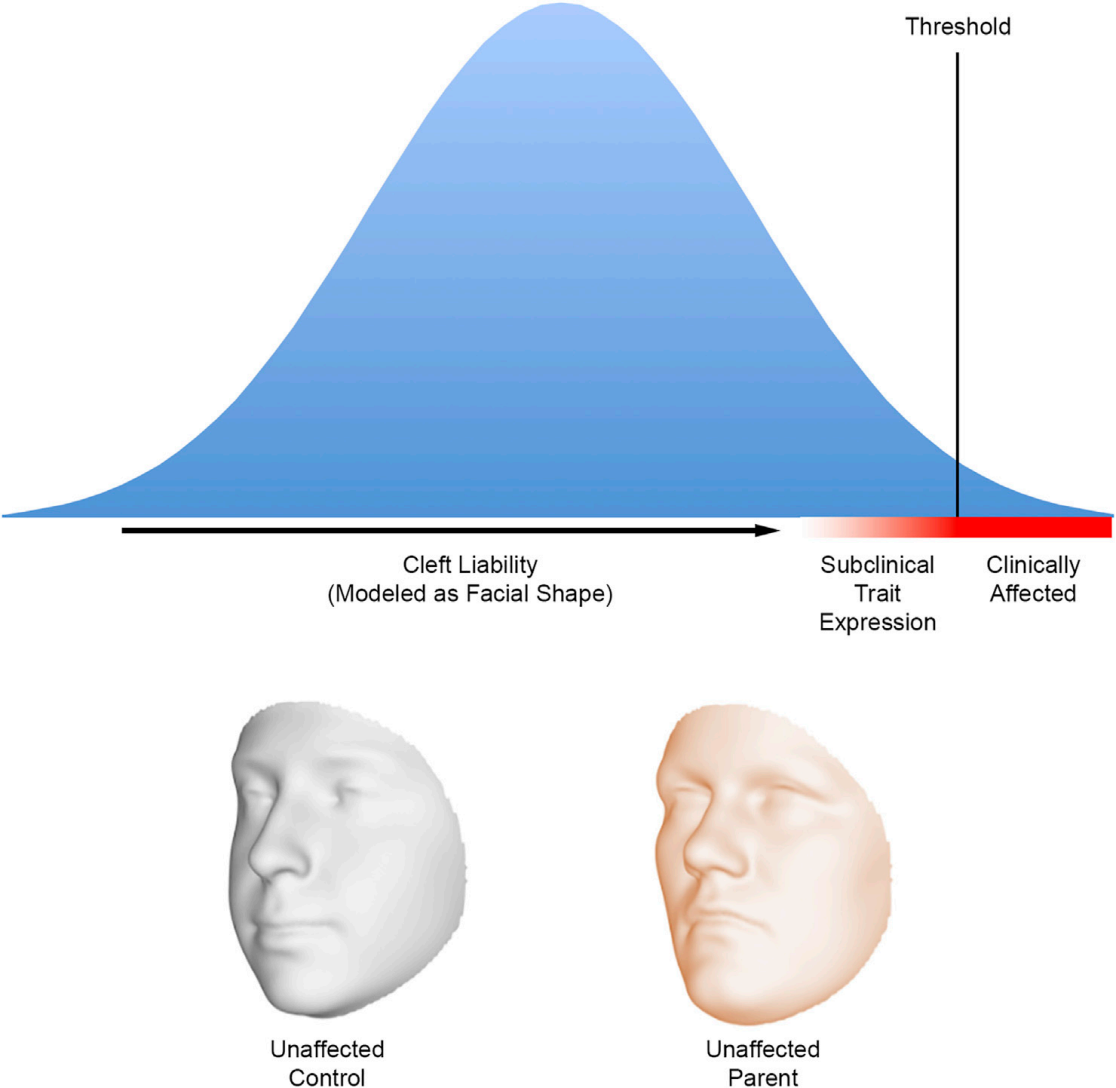
A hypothetical liability threshold model for orofacial clefting where the liability is modelled as facial shape. The facial shape of individuals near the defect threshold is expected to differ on average from the general population; the altered facial shape can be considered a subclinical expression resulting from the presence of cleft risk factors. The biological relatives of those affected with orofacial clefts are expected to be closer to the threshold—and therefore exhibit facial differences—compared with an individual chosen at random from the population. The faces below the graph show how the 3D facial surface changes shape from a hypothetical control to a hypothetical unaffected parent of a child affected by nonsyndromic CL/P. These 3D morphs are based on a comparison of 264 parental faces to 3,171 control faces (Indencleef et al., 2021). Note the relative midfacial flattening and upper facial broadening in the parental face. Faces adapted from Indencleef et al. (2021), CC BY 4.0.

The multifactorial threshold (MFT) concept emerged in the 1930’s as a way to model the inheritance of discrete traits in mammals that did not follow strict Mendelian patterns (Wright, 1934a; 1934b). Application of the MFT model to human diseases and malformations, including orofacial clefting, began in the 1960’s with the work of Falconer, Carter, and Fraser (Curnow and Smith, 1975). The MFT model offered a way to conceptualize liability as a continuum and make predictions about recurrence. For clefting, the nature of what might underlie the liability, and threshold has been a matter of considerable debate (Fraser, 1976). In the context of the FSH, the MFT model provided a conceptual framework for prior research showing altered facial features in at-risk mouse strains and families with a history of orofacial clefting. Facial shape could now be considered one of the factors underlying cleft liability. Because seemingly unaffected family members possess at least some the same genetic risk factors as their affected relatives, they should be on average closer to the threshold than the rest of the population (Figure 1). As a result of their position on the liability continuum, we then expect these relatives to manifest craniofacial differences compared to the general population (Fraser, 1970).

## Over 50 Years of Measuring Faces

By the start of the 1970’s, the notion that certain aspects of facial shape may contribute to cleft liability was beginning to gain acceptance. Fraser and Pashayan (1970) published a widely cited study comparing the facial dimensions of non-cleft parents with affected children to controls. Using several measurement approaches, they described a suite of facial differences in parents, including increased upper facial breadth, increased facial length, and reduced maxillary projection. The notion that quantitative facial differences will be present in the unaffected members of families with a history of clefting is based on a few assumptions: 1) that facial morphology can be reliability quantified; 2) that the relevant facial phenotypes are at least partly heritable; and 3) that alterations in embryonic facial shape are retained and can be detected postnatally. The first two assumptions are well supported, while the third is not plausible to test in humans, but is supported in mice (Boughner et al., 2008).

Since these early efforts, there have been over 30 quantitative studies comparing facial morphology between the ostensibly unaffected parents and/or siblings of individuals with clefts and controls with no family history of the defect. These studies employ a variety of methods including cephalometry (Coccaro et al., 1972; Kurisu et al., 1974; Shibasaki et al., 1978; Nakasima and Ichinose, 1983, 1984; Procházková and Tolarová, 1986; Sato, 1989; Ward et al., 1989, 1994; Raghavan et al., 1994; Procházková and Vinšová, 1995; Laatikainen et al., 1996; Mossey et al., 1998; AlEmran et al., 1999; Suzuki et al., 1999; McIntyre and Mossey, 2003; Perkiomaki et al., 2003; Chatzistavrou et al., 2004; McIntyre and Mossey, 2004; Yoon et al., 2004), direct anthropometry (Figalová and Šmahel, 1974; Blanco et al., 1992), 2D photogrammetry (Erickson, 1974) and 3D surface imaging (Weinberg et al., 2008, 2009; Miller et al., 2014; Roosenboom et al., 2017; Indencleef et al., 2021). A distinct subset of these studies is also focused on measuring patterns of craniofacial asymmetry in at-risk relatives (Pashayan and Fraser, 1971; Farkas and Cheung, 1979; Sigler and Ontiveros et al., 1999; McIntyre and Mossey, 2002a, 2010; Yoon et al., 2003; Kumar et al., 2010; Miller et al., 2014; Zhang et al., 2018). These studies cover a diverse array of populations, including individuals of East Asian, European, and Latin American Admixed ancestry. It is neither practical, nor worthwhile, to review every one of these studies. One reason is that they are so heterogeneous in terms of morphometric approach, sampling, and inclusion criteria that it is difficult to draw meaningful comparisons.

What we can state is that every study conducted thus far has reported at least some significant differences in facial morphology between unaffected relatives and controls. Moreover, these differences have been reported for every part of the craniofacial complex and many appear to be sex-specific. However, sorting out which specific facial features are reliable indicators of cleft-risk has been a challenge. There have been a few narrative reviews on the topic (McIntyre and Mossey, 2002b; Ward et al., 2002; Maulina et al., 2006), but it is difficult to avoid biased conclusions when the literature is rife with conflicting results. In attempt to get at something of a consensus, Weinberg et al. (2006) performed a meta-analysis of the parent versus control cephalometric literature available up to that point. After analyzing 28 measures, they concluded that the following suite of facial differences best characterized unaffected parents of children with CL/P: wider interorbital, nasal cavity and upper facial dimensions; narrower cranial vaults; longer cranial bases; longer and more protrusive mandibles; vertically shorter upper faces; and vertically longer lower faces. While these results still need to be considered with caution due to the small number of studies that met the inclusion criteria, it is worth noting that some of the traits identified—most notably a tendency toward increased midface retrusion and broader mid and upper facial dimensions—have now been validated by more recent studies using advanced 3D surface imaging and geometric morphometric techniques to assess biological shape (Weinberg et al., 2008, 2009; Indencleef et al., 2021). These differences are shown in Figure 1. Perhaps ironically, this morphological pattern echoes the original findings of Fraser and Pashayan from over 50 years ago.

## THE Genetic Intersection Between Face Shape and Clefting

One expectation of the FSH is that the genes that drive face shape variation in humans will also be implicated in orofacial clefting (and vice-versa). Boehringer et al. (2011) tested this claim in two non-cleft European cohorts by evaluating the relationship between facial measures shown previously to differ between at-risk relatives and controls (nasal width and upper facial width) and 11 SNPs previously identified from orofacial clefting association studies. The result: two SNPs showed significant but weak genotype-phenotype relationships. A year later, in one of the first genome-wide association studies (GWAS) of normal-range facial morphology, Liu et al. (2012) performed targeted tests involving these same 11 candidate SNPs and 48 different facial measures; they reported significant associations at four loci involving horizontal upper facial dimensions. More recently, Indencleef et al. (2018) tested a set of 65 SNPs previously implicated in human studies of nonsyndromic clefting to determine their effects on normal-range 3D facial shape. They reported positive evidence for six SNPs including those near *NOG* (associated with philtrum shape), *THADA* (associated with supraorbital ridge morphology) and *PAX7*, *MSX1*, and *PTCH1* (all associated with nose shape).

**FIGURE 2:**
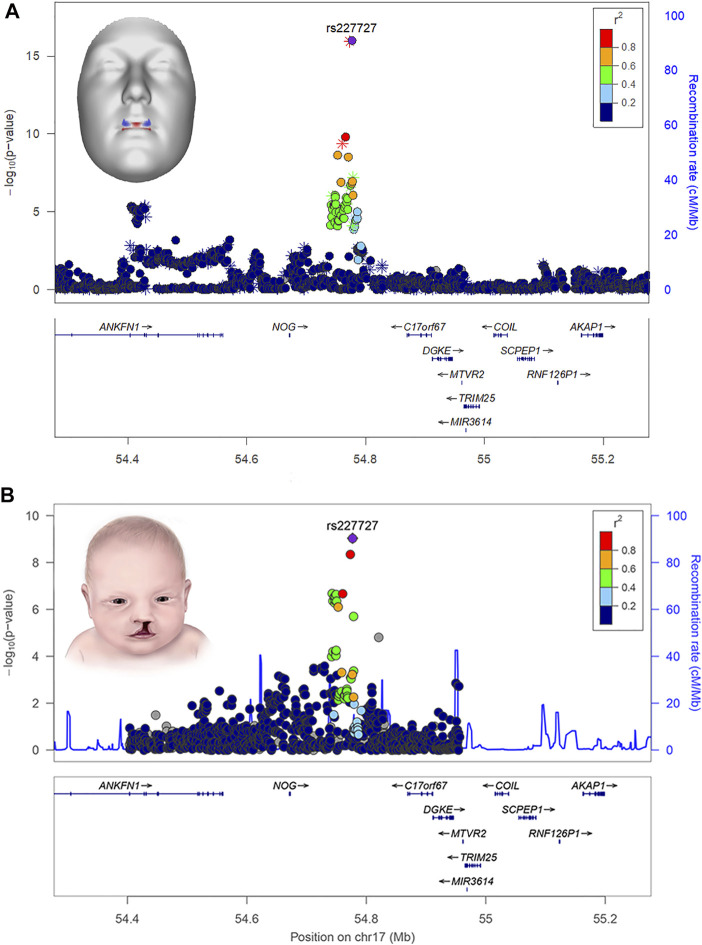
LocusZoom plots showing the statistical association of SNP rs227727 near the gene *NOG* with lip shape **(A)** and nonsyndromic orofacial clefting **(B)**. The 3D face inset for panel A (top) shows the effects of the SNP as color-coded heat map where red indicates regions of the face moving in an outward direction and blue indicates regions of the face moving in an inward direction. The lip shape association was reported originally in White et al. (2021). The cleft association was reported originally in Leslie et al. (2015). Attribution: the inset for panel A (top) was adapted from White and Indencleef, Insights into the genetic architecture of the human face, via Figshare, CC BY 4.0, https://doi.org/10.6084/m9.figshare.c.4667261.v6. The inset for panel B (bottom) is from the Centers for Disease Control and Prevention and is available under the Creative Commons CC0 1.0 Universal Public Domain Dedication, via Wikimedia Commons, https://commons.wikimedia.org/wiki/File:Cleftlip.jpg.

Several human face shape GWASs have now been published (Naqvi et al., in press) and many of the chromosomal regions identified have been found to harbor genes implicated in orofacial clefting and/or palate formation. To illustrate, in one of the most comprehensive facial GWAS to date, White et al. (2021) scanned the genomes of over 8,000 individuals and identified 203 regions across the genome associated with aspects of normal-range 3D facial shape. Bioinformatic analysis of the implicated genes revealed significant enrichment (i.e., statistical over-representation) for phenotypes and developmental processes directly related to clefting; some of the implicated cleft candidate genes included *PRDM16*, *PAX7*, *PKDCC*, *SATB2*, *COL8A1*, *MSX1*, *FAT4*, *FGFR2*, *PAX9*, *BMP4*, *GREM1*, *NOG*, *BMP2*, *PAX1*, and *BMP7*. Moreover, in many cases, phenotypic effects at the cleft-related GWAS signals were localized to anatomical regions directly relevant to clefting, like the central midface. For example, the rs227727 SNP near *NOG* was strongly associated with normal-range upper lip shape (Figure 2). Notably, this same variant was previously identified as functionally relevant for nonsyndromic CL/P (Leslie et al., 2015). In another example, *PAX1* was associated with normal-range morphology of the nasal alae and philtrum and was recently shown to impact risk of cleft subtypes involving only of the lip (Curtis et al., 2021).

Leveraging existing data, Howe et al. (2018) investigated the shared genetics between facial shape and orofacial clefting by constructing a polygenic risk score (PRS) for clefting and testing whether that PRS was associated with selected facial dimensions previously shown to be relevant for cleft risk from family studies. They found that the cleft PRS was significantly associated with decreased philtrum width and, using Mendelian randomization, showed that this relationship was causal (i.e., genetic variants for clefting cause decreased philtrum width). Indencleef et al. (2021) also investigated the question of genetic overlap starting with an improved delineation of the cleft-relevant facial features. Using a highly innovative facial phenotyping approach that naturally breaks the 3D facial geometry into hieratically arranged regions (Claes et al., 2018), the authors explicitly measured aspects of 3D facial morphology that differed between at-risk relatives and controls. Once identified, the shape phenotypes indicative of elevated cleft risk were then measured in a sample of over 8,000 healthy individuals with available 3D facial and genomic data. A series of GWASs were then performed to better understand the genetic architecture of these cleft risk traits, resulting in the identification of 29 loci. Twenty-two of these loci were previously associated with normal facial variation and 18 were near genes known to play a role in orofacial clefting. Moreover, a PRS for nonsyndromic orofacial clefting was statically associated with several of the identified facial risk traits.

As noted earlier, strains of mice with high and low susceptibility to clefting show differences in facial shape. For example, Parsons et al. (2008) showed that cleft-susceptible A/WySn embryos had underdeveloped and more divergently spaced facial maxillary prominences compared with C57BL/6J embryos. In recent years, a picture has started to emerge regarding the molecular factors driving these and other shape changes associated with increased risk of clefting. For example, Lipinski et al. (2010) showed that interfering with *sonic hedgehog* expression early in facial formation results by gestational day (GD) 11.25 in mispositioned and misshapen medial nasal prominences that can no longer make contact to form an intact primary palate. When examined at a slightly later timepoint (GD 17), treated mice showed midface hypoplasia and broader upper facial dimensions, and many developed clefts (Lipinski et al., 2014). In another study, *Tfap2a* mutant mouse embryos that develop CL/P were shown at E10.5 and E11.5 to have misaligned and misshaped nasal and maxillary prominences with reduced cell proliferation, driven in part by aberrant Fgf signaling (Green et al., 2015). In a later study, Green et al. (2019) showed that the cleft-related shape changes evident in the A/WySn embryonic face are linked to the degree of *Wnt9b* methylation and expression. Taken together, these studies point to specific genes and pathways driving changes in facial shape and variation that can push individuals along the liability spectrum toward the threshold, leading to an increased risk of clefting.

## Impact and Future Outlook

Our knowledge connecting facial shape to orofacial clefting has evolved from the clinical anecdotes of 19^th^ century surgeons to modern experiments using the latest imaging, molecular and bioinformatic techniques. However, translating this knowledge into clinically meaningful and actionable information remains a significant challenge. As stated earlier, we know that different kinds of mechanisms can lead to clefts during early facial formation. Lumping all orofacial clefts together as a monolithic group ignores these differences, which can have implications for counseling, scientific discovery, and even treatment. The aspiration is that investigating patterns of facial shape in families can be used to help sort though some of this etiological heterogeneity and build a more complete picture of the genetic players involved in conferring orofacial cleft risk. Even a goal as crude as classifying individuals and their families into broad etiological classes based on whether the cleft was impacted by facial shape may prove useful. In the realm of genetic counseling, such knowledge could potentially lead to greater precision and personalization in cleft recurrence risk estimation. From the perspective of scientists invested in uncovering the genetic architecture of orofacial clefts, this type of phenotypic refinement has the potential to boost discovery power. Perhaps we can envision building a robust PRS for clefting, informed by genetic variants known to impact aspects of facial shape related to cleft risk. Studies investigating the genetic basis of facial risk phenotypes (e.g., Indencleef et al., 2021) can reveal variants that play a role in orofacial clefting, but whose effects may be too small to be detected in a standard cleft case-control GWAS. Such variants are likely to be modifiers of cleft risk, each with a small individual effect, but enough to nudge individuals toward the defect threshold or perhaps toward a more severe phenotypic expression. To illustrate this concept, Yu et al. (2022) showed that common genetic risk factors for clefting (modeled as a PRS) can modify the phenotypic penetrance of a rare damaging mutation in family with a history of nonsyndromic CL/P.

These problems are complicated and will require considerable effort to resolve. More immediately, though, there are still some basic questions about face shape and clefting that need to be investigated. For example, we have a poor understanding of whether the facial features associated with cleft predisposition differ according to the type of cleft(s) present in families (e.g., CL/P versus CPO), although at least one study has shown differences along these lines (Mossey et al., 1997). Similarly, we don’t know how the facial patterns observed in unaffected relatives compare across different ancestral backgrounds or whether any ancestry-specific patterns might help explain differences in cleft incidence among human populations. This was suggested by Chung and Kau (1985), who showed a relationship between specific facial dimensions and cleft predisposition by comparing individuals from various ancestral backgrounds (and therefore cleft prevalences) residing in a single location. Even the long-recognized sex biases we see in orofacial clefting may relate to sex differences in embryonic facial development, as initially suggested by Burdi and Silvey (1969). We know that sex differences in human facial morphology are present across the lifespan (Kesterke et al., 2016; Matthews et al., 2018) and that many of the facial differences we see in unaffected parents are sex-specific (Weinberg et al., 2008). Nevertheless, the data connecting sex, face shape, and cleft predisposition remains speculative. To answer these kinds of questions, we will undoubtedly need data from larger and more diverse samples, which should be a priority for future investigations.
